# The retrieval of neutral and positive autobiographical memories: a pilot study

**DOI:** 10.12688/f1000research.146863.1

**Published:** 2024-04-17

**Authors:** Xinning Su, Akira Midorikawa

**Affiliations:** 1 Department of Psychology, Chuo University, Tokyo, Japan

**Keywords:** autobiographical memories, emotional valence, positive memory bias, neutral–positive memories

## Abstract

**Background:**

Several studies have shown that the retrieval of positive memories may play a role in emotional regulation. However, it is unclear whether the effects of neutral and positive autobiographical memories differ. There is a need to confirm that genuinely neutral autobiographical memories can be retrieved without bias in response to a prompt to recall neutral memories.

**Methods:**

In this pilot study, using “home” and “study” as cue words, we investigated whether participants were able to retrieve appropriate autobiographical memories when asked to recall a limited number of neutral or positive memories.

**Results:**

Although participants were asked to recall neutral autobiographical memories, they tended to recall positive memories.

**Conclusions:**

Our results support the concept of positive memory bias and suggest that future work should consider combining neutral and positive autobiographical memories, namely by asking participants to recall neutral–positive memories.

## Introduction

Autobiographical memory is defined as memory for information relating to the self ( Brewer, 1986). It is believed to play a role in shaping an individual’s self-awareness and identity over time ( Conway, 2005; Conway & Pleydell-Pearce, 2000; Tulving, 2002). Autobiographical memory is strongly linked to emotion ( Holland & Kensinger, 2010). Several studies have shown that individuals with emotional disorders, such as depression ( King et al., 2010) and anxiety ( Krans et al., 2014), exhibit autobiographical memory biases; for example, they exhibit more negative autobiographical memories compared with control participants.

Although individuals may repeatedly recall past negative experiences to learn from their mistakes, repeated recall of negative events constitutes rumination, a maladaptive cognitive process that is strongly associated with the onset and perpetuation of depression ( Nolen-Hoeksema & Morrow, 1991; Nolen-Hoeksema et al., 2008). Harris et al. (2010) found that repeated retrieval of positive autobiographical memories inhibited the recall of negative autobiographical memories associated with the same cue word. Additionally, Burton and King (2004, 2009) found that writing about positive experiences increased positive affect. These results suggest that the retrieval of positive memories can facilitate emotional regulation.

In addition to autobiographical memories with positive or negative valence, memories can be neutral ( D’Argembeau et al., 2003). It is unclear whether neutral and positive autobiographical memories contribute in a similar manner to emotion regulation. However, before that question can be answered, there is a need to determine whether genuinely neutral autobiographical memories can be retrieved without bias in response to a prompt to recall neutral memories. Several studies have shown that when participants are asked to recall as many autobiographical memories as possible, they generally recall more positive autobiographical events than neutral and negative events ( Berntsen, 1996; Clark et al., 2013; Marsh et al., 2019). This phenomenon is known as positive memory bias ( Adler & Pansky, 2020; Skowronski, 2011; Walker et al., 2003). However, most previous studies did not limit the number of recalls, which varied among events according to emotional valence. Notably, it is unclear whether participants can recall genuinely neutral memories when prompted if the number of recalls is limited.

Therefore, in this pilot study, we adopted the cue words used by Harris et al. (2010), namely “home” and “study”, to investigate whether appropriate autobiographical memories can be retrieved by participants who are asked to recall a limited number of neutral or positive memories.

## Methods

### Participants and study design

In total, 10 students (five men and five women) participated in this study (mean age = 21.90 years; range: 19–24 years). All participants were enrolled in a preparatory school in Tokyo, Japan, and recruited in a classroom setting.

The experiment had a 2 × 2 mixed design. The between-subjects factor was valence (positive/neutral), and the within-subjects factor was cue (home/study).

### Ethics approval and informed consent

This study was conducted in line with the Declaration of Helsinki. It was approved by the ethics committee of Chuo University, on 4 August 2020, vide approval number 2020-02. Written informed consent was obtained from all individual participants included in the study.

### Procedure

Participants were divided into positive and neutral groups. The two cue words, home and study, were presented to each group. Participants were asked to generate five positive or five neutral autobiographical memories for each cue. Researchers informed the participants that the “study events” were associated with study or work, whereas the “home events” were associated with home or family.

After the retrieval of autobiographical memories for each cue word, participants briefly reported the context of the recalled events in a verbal manner, and a researcher recorded the description. After they had reported events, participants were asked to rate the valence of those events (1 = *Very negative*, 7 = *Very positive*).

## Results

### One-sample t-tests

**Figure 1.**
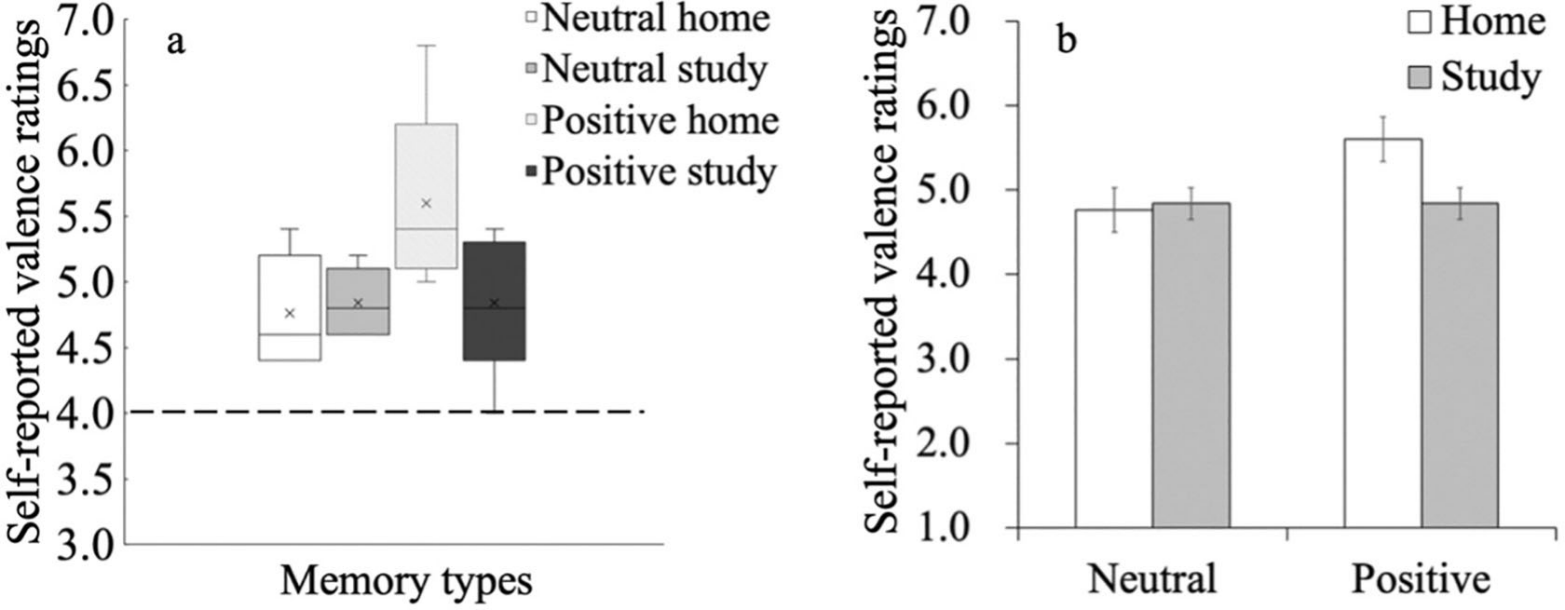
(a) Box-and-whisker plot for the four memory types and (b) and self-reported valence ratings. Dashed line in (a) is the neutral score (baseline), and error bars in (b) represent standard error.

On a 7-point Likert response scale, the neutral score (i.e., 4) was regarded as the baseline; differences between self-rated scores for the four memory types (i.e., home memories in the positive group, study memories in the positive group, home memories in the neutral group, and study memories in the neutral group) and the neutral score were calculated. One-sample t-tests were used to determine whether differences between the scores for each memory type and the neutral score were significant. We found significant differences between scores for all memory types and the neutral score (home memory in the positive group, *t*_(4)_ = 5.06, *p* < 0.01, *d* = 2.26; study memory in the positive group, *t*_(4)_ = 3.50, *p* = 0.03, *d* = 1.57; home memory in the neutral group, *t*_(4)_ = 3.92, *p* = 0.02, *d* = 1.75; and study memory in the neutral group, *t*_(4)_ = 7.20, *p* < 0.01, *d* = 3.22) ( Figure 1a).

### Analysis of variance (ANOVA)

As shown in Figure 1b, a 2 (valence group: positive/neutral) × 2 (cue: home/study) mixed ANOVA for event valence was conducted. There was no main effect of valence group ( *F*_(1,8)_ = 3.30, *p* = 0.11, $\eta_{p}^{2}$ = 0.29), no main effect of cue ( *F*_(1,8)_ = 2.27, *p* = 0.17, $\eta_{p}^{2}$ = 0.22), and no interaction effect ( *F*_(1,8)_ = 3.35, *p* = 0.10, $\eta_{p}^{2}$ = 0.30).

## Discussion

In this study, we used the cue words home and study to determine whether appropriate autobiographical memories could be retrieved by participants who were asked to recall neutral or positive memories. We found a bias toward recalling positive events in both the neutral and positive groups, although participants were asked to recall neutral events. Moreover, there were no significant differences in memory ratings between the two groups.

One explanation for the above results is positive memory bias ( Adler & Pansky, 2020; Skowronski, 2011; Walker et al., 2003). Several studies have shown that more positive autobiographical events are generally recalled, whether voluntarily or involuntarily, compared with neutral or negative events ( Berntsen, 1996; Clark et al., 2013; Marsh et al., 2019). However, unlike previous studies, we limited the number of recalled memories, asking participants to generate five positive or five neutral autobiographical memories for each cue. Thus, the neutral and positive groups did not differ according to the number of recalls; nevertheless, they showed emotional valence bias. The self-memory system, in which autobiographical memory retrieval is influenced by current beliefs, active goals, and self-image, supports positive memory bias ( Conway, 2005). People generally retrieve autobiographical memories consistent with their self-image and beliefs, which tend to be positive.

In conclusion, although participants in the present study were asked to recall neutral autobiographical memories, they tended to recall positive events. Future work should consider combining neutral and positive autobiographical memories, namely by asking participants to recall neutral–positive memories. We anticipate more studies investigating whether, similar to the effects of retrieving positive memories ( Burton & King, 2004; Burton & King, 2009; Harris et al., 2010), the retrieval of neutral–positive memories also inhibits the recall of negative memories or increases positive emotions. Such studies may inform strategies for emotional regulation.

### Ethics approval and informed consent

This study was conducted in line with the Declaration of Helsinki. It was approved by the Ethics Committee of Chuo University, on 4 August 2020, vide approval number 2020-02. Written informed consent was obtained from all individual participants included in the study.

## Data Availability

This study contains the following underlying data: Fighsare, Data for “the retrieval of neutral and positive autobiographical memories: a pilot study” https://doi.org/10.6084/m9.figshare.24939264.v1 ( Su et al., 2024). Data are available under the terms of the Creative Commons Attribution 4.0 International license (CC-BY 4.0).
