# The retrieval of neutral and positive autobiographical memories: a pilot study

**DOI:** 10.12688/f1000research.146863.2

**Published:** 2024-10-22

**Authors:** Xinning Su, Akira Midorikawa

**Affiliations:** 1 Department of Psychology, Chuo University, Tokyo, Japan

**Keywords:** autobiographical memories, emotional valence, positive memory bias, neutral–positive memories

## Abstract

**Background:**

Several studies have shown that the retrieval of positive memories may play a role in emotional regulation. However, it is unclear whether the effects of neutral and positive autobiographical memories differ. It is necessary to confirm whether genuinely neutral autobiographical memories can be retrieved without bias when prompted to recall neutral memories.

**Methods:**

In this pilot study, using “home” and “study” as cue words, we investigated whether participants were able to retrieve appropriate autobiographical memories when asked to recall a limited number of neutral or positive memories.

**Results:**

Although participants were asked to recall neutral autobiographical memories, they tended to recall positive memories.

**Conclusions:**

Our results support the concept of positive memory bias and suggest that future work should consider combining neutral and positive autobiographical memories by asking participants to recall neutral–positive memories.

## Introduction

Autobiographical memory is defined as memory for information relating to the self ( Brewer, 1986). It is believed to play a role in shaping an individual’s self-awareness and identity over time ( Conway, 2005; Conway & Pleydell-Pearce, 2000; Tulving, 2002). Autobiographical memory is strongly linked to emotion ( Holland & Kensinger, 2010). Several studies have shown that individuals with emotional disorders, such as depression ( King et al., 2010) and anxiety ( Krans et al., 2014), exhibit autobiographical memory biases. For example, these individuals tend to recall more negative autobiographical memories compared to control participants. While individuals may recall past negative experiences to learn from their mistakes, excessive recall of negative events often leads to rumination, a maladaptive cognitive process associated with the onset and perpetuation of depression ( Nolen-Hoeksema & Morrow, 1991; Nolen-Hoeksema et al., 2008). This suggests a need for strategies to mitigate the negative impact of such memories.

Harris et al. (2010) found that repeated retrieval of positive autobiographical memories can lead to forgetting negative memories associated with the same cue word. This finding implies that positive memory retrieval can inhibit the recall of unwanted negative autobiographical memories. However, in addition to autobiographical memories with positive or negative valence, memories can also be neutral ( D’Argembeau et al., 2003). Regarding whether neutral memory retrieval can also inhibit the recall of negative memories, evidence from recent studies on semantic memory indicates that retrieving neutral semantic memories can lead to forgetting related negative memories ( Greer et al., 2024). Considering that autobiographical and semantic memories share overlapping neural bases ( Graham et al., 2003), and negative autobiographical memories are more strongly associated with emotional disorders ( King et al., 2010; Krans et al., 2014), it is worth investigating whether the effects observed with neutral semantic memories might also apply to neutral autobiographical memories. Should neutral autobiographical memories function similarly to positive ones, this could extend the findings of Harris et al. (2010), offer new insights into the mechanisms of memory and emotion, and suggest alternative strategies for mitigating the effects of negative memories.

However, before we extend the findings of Harris et al. (2010), a pilot study is necessary to determine whether genuinely neutral autobiographical memories can be retrieved without bias when prompted to recall neutral memories. Previous studies have shown that participants tend to recall more positive autobiographical events when asked to recall as many memories as possible ( Berntsen, 1996; Clark et al., 2013; Marsh et al., 2019). Additionally, recall order is influenced by the emotional content of memories, with emotional memories often recalled before neutral ones ( Nusser & Zimprich, 2021; Zimprich & Nusser, 2023). This phenomenon, known as positive memory bias ( Adler & Pansky, 2020; Skowronski, 2011; Walker et al., 2003), suggests that neutral memories may not be the primary focus unless specifically cued. The self-memory system ( Conway, 2005) suggests that autobiographical memory retrieval is influenced by an individual’s current beliefs, active goals, and self-image, which tend to be positive. As a result, people may unconsciously gravitate toward recalling positive autobiographical memories.

However, most previous studies did not limit the number or valence of recalls. According to the procedure of Harris et al. (2010), participants were explicitly asked to recall a limited number of specified emotional events associated with each cue word. Since our ultimate aim is to extend the findings of Harris et al. (2010), in the present pilot study, we adopted the same cue words as Harris et al. (2010), namely “home” and “study”, to investigate whether appropriate autobiographical memories when asked to recall a limited number of neutral or positive memories.

## Methods

### Participants and study design

In total, 10 students (five men and five women) participated in this study (mean age = 21.90 years; range: 19–24 years). All participants were enrolled in a preparatory school in Tokyo, Japan, and were recruited in a classroom setting.

The experiment had a 2 × 2 mixed design. The between-subjects factor was valence (positive/neutral), and the within-subjects factor was cue (home/study).

### Ethics approval and informed consent

This study was conducted in line with the Declaration of Helsinki. It was approved by the ethics committee of Chuo University, on 4 August 2020, vide approval number 2020-02. Written informed consent was obtained from all individual participants included in the study.

### Procedure

Participants were divided into positive and neutral groups. The cue words used by Harris et al. (2010) were “home” and “work”, likely chosen due to the wide age range of their participants (17–42 years old). For the cue word “work”, Harris et al. (2010) asked participants to recall autobiographical memories related to study or work. Given that all of our participants were college students of similar age, the cue words in our experiment were “home” and “study”, presented to each group. Participants were asked to generate five positive or five neutral autobiographical memories for each cue. Researchers informed the participants that the “study events” should be associated with study or work, whereas the “home events” should be related to home or family.

After retrieving autobiographical memories for each cue word, participants briefly reported the context of the recalled events verbally, and a researcher recorded their descriptions. Then, as in Harris et al.’s (2010) methodology, after they had reported the events, participants were asked to rate the valence of those events (1 = *Very negative*, 7 = *Very positive*).

## Results

### One-sample t-tests

**Figure 1.**
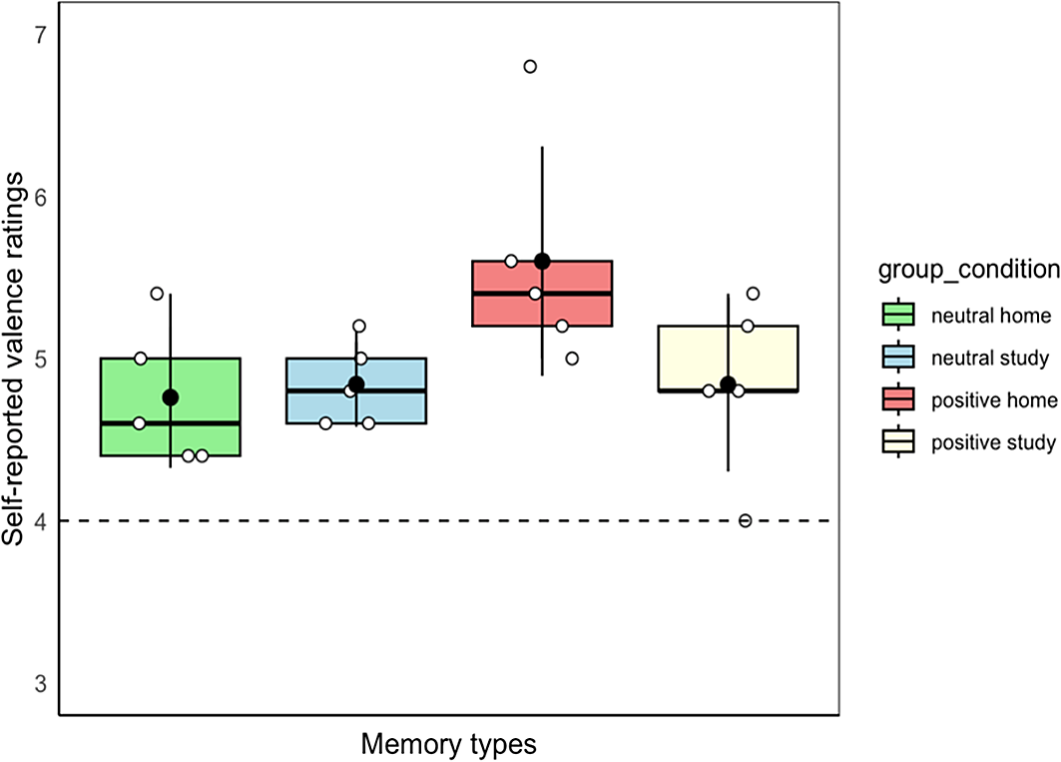
Box-and-whisker plot for the four memory types. Dashed line is the neutral score (baseline), and error bars represent standard deviation.

On a 7-point Likert response scale, the neutral score (i.e., 4) was regarded as the baseline; differences between self-rated scores for the four memory types (i.e., home memories in the positive group, study memories in the positive group, home memories in the neutral group, and study memories in the neutral group) and the neutral score were calculated. One-sample t-tests were used to determine whether differences between the scores for each memory type and the neutral score were significant. We found significant differences between scores for all memory types and the neutral score (home memories in the positive group, *t*_(4)_ = 5.06, *p* < 0.01, *d* = 2.26; study memories in the positive group, *t*_(4)_ = 3.50, *p* = 0.03, *d* = 1.57; home memories in the neutral group, *t*_(4)_ = 3.92, *p* = 0.02, *d* = 1.75; and study memories in the neutral group, *t*_(4)_ = 7.20, *p* < 0.01, *d* = 3.22) ( Figure 1).

### Analysis of variance (ANOVA)

As shown in Figure 1, a 2 (valence group: positive/neutral) × 2 (cue: home/study) mixed ANOVA for event valence was conducted. There was no main effect of valence group ( *F*_(1,8)_ = 3.30, *p* = 0.11, $\eta_{p}^{2}$ = 0.29), no main effect of cue ( *F*_(1,8)_ = 2.27, *p* = 0.17, $\eta_{p}^{2}$ = 0.22), and no interaction effect ( *F*_(1,8)_ = 3.35, *p* = 0.10, $\eta_{p}^{2}$ = 0.30).

## Discussion

In this study, we used the cue words “home” and “study” to determine whether appropriate autobiographical memories could be retrieved by participants who were asked to recall a limited number of neutral or positive memories. We found a bias toward recalling positive events in both the neutral and positive groups, even though participants were asked to recall neutral events. Moreover, there were no significant differences in memory ratings between the two groups.

One explanation for these results is positive memory bias ( Adler & Pansky, 2020; Skowronski, 2011; Walker et al., 2003). The self-memory system ( Conway, 2005) suggests that autobiographical memory retrieval is influenced by people’s current beliefs, active goals, and self-image, which tend to be positive. As a result, people may unconsciously tend to recall positive autobiographical memories. Several studies have shown that more positive autobiographical events are generally recalled, whether voluntarily or involuntarily, compared with neutral or negative events ( Berntsen, 1996; Clark et al., 2013; Marsh et al., 2019). However, unlike previous studies, we limited the number of recalled memories, asking participants to generate five positive or five neutral autobiographical memories for each cue. Thus, the neutral and positive groups did not differ in the number of recalls; nevertheless, they showed emotional valence bias.

This study had some limitations. First, since our ultimate aim is to extend the findings of Harris et al. (2010), the methods and cue words employed in this pilot study align with those described by Harris et al. (2010). While this approach is appropriate for the present study’s purposes, it would be beneficial to incorporate more neutral cue words to broaden the results further. Second, we adopted self-rating valence as per the methodology of Harris et al. (2010). However, self-ratings may be biased by various factors, including participants’ subjective interpretation of what constitutes a neutral event and their current mood ( Chiorri & Vannucci, 2024). Given the potential for subjective bias, future studies should incorporate both subjective self-ratings and objective assessments by independent raters to ensure a more accurate categorization of recalled events. This will allow for better verification of whether neutral memories are indeed perceived as neutral or if a bias toward positivity has occurred.

In conclusion, despite some limitations, we found that when limiting the number of recalls, participants tended to recall positive events even when asked to recall neutral autobiographical memories. Previous studies have found that retrieval of neutral semantic memories leads to forgetting of negative semantic memories ( Greer et al., 2024), while retrieval of positive autobiographical memories leads to forgetting of negative autobiographical memories ( Harris et al., 2010). Therefore, it is necessary to extend the findings of Harris et al. (2010) to examine whether neutral autobiographical memories can also inhibit the recall of negative memories. However, before extending the findings of Harris et al. (2010), we conducted this pilot study to determine whether genuinely neutral autobiographical memories can be retrieved without bias when prompted to recall neutral memories. Based on the results of this pilot study, it will be necessary to continue refining our understanding. Additionally, we can propose innovative ideas, such as exploring whether future work could integrate neutral and positive autobiographical memories, by asking participants to recall neutral–positive memories. We look forward to further research investigating whether, similar to the effects of retrieving positive memories ( Burton & King, 2004; Burton & King, 2009; Harris et al., 2010), retrieving neutral–positive memories may also inhibit the recall of negative memories or enhance positive emotions. Such studies could provide valuable insights for developing emotional regulation strategies.

### Ethics approval and informed consent

This study was conducted in line with the Declaration of Helsinki. It was approved by the Ethics Committee of Chuo University, on 4 August 2020, vide approval number 2020-02. Written informed consent was obtained from all individual participants included in the study.

## Data Availability

This study contains the following underlying data: Fighsare, Data for “the retrieval of neutral and positive autobiographical memories: a pilot study” https://doi.org/10.6084/m9.figshare.24939264.v1 ( Su, 2024). Data are available under the terms of the Creative Commons Attribution 4.0 International license (CC-BY 4.0).
