# Supplementary material for: The retrieval of neutral and positive autobiographical memories: a pilot study
Source: F1000Res. 2024 Oct 22;13:285. Originally published 2024 Apr 17. [Version 2] doi: 10.12688/f1000research.146863.2 (PMC11635190; doi:10.12688/f1000research.146863.2)
Supplement: Peer review history

# Reviewer report: Reviewer response for version 2

**DOI:** 10.5256/f1000research.173083.r333848

**Published:** 2024-12-11

**Authors:** Arkadiy Maksimovskiy

**Affiliations:** 1 Howard Hughes Medical Institute - Harvard Medical School, Boston, Massachusetts, USA

All comments were successfully addressed.

Is the work clearly and accurately presented and does it cite the current literature?

Yes

If applicable, is the statistical analysis and its interpretation appropriate?

No

Are all the source data underlying the results available to ensure full reproducibility?

Yes

Is the study design appropriate and is the work technically sound?

Partly

Are the conclusions drawn adequately supported by the results?

Partly

Are sufficient details of methods and analysis provided to allow replication by others?

Yes

Reviewer Expertise:

Cognitive neuroscience and memory research.

I confirm that I have read this submission and believe that I have an appropriate level of expertise to confirm that it is of an acceptable scientific standard.

# Reviewer report: Reviewer response for version 2

**DOI:** 10.5256/f1000research.173083.r333847

**Published:** 2024-11-04

**Authors:** Xinxu Shen

**Affiliations:** 1 Temple University, Philadelphia, Pennsylvania, USA

I think the revised version is good for indexing.

Is the work clearly and accurately presented and does it cite the current literature?

Yes

If applicable, is the statistical analysis and its interpretation appropriate?

Partly

Are all the source data underlying the results available to ensure full reproducibility?

Yes

Is the study design appropriate and is the work technically sound?

Partly

Are the conclusions drawn adequately supported by the results?

Partly

Are sufficient details of methods and analysis provided to allow replication by others?

Yes

Reviewer Expertise:

episodic memory, decision making

I confirm that I have read this submission and believe that I have an appropriate level of expertise to confirm that it is of an acceptable scientific standard.

# Reviewer report: Reviewer response for version 1

**DOI:** 10.5256/f1000research.160988.r298790

**Published:** 2024-07-29

**Authors:** Xinxu Shen

**Affiliations:** 1 Temple University, Philadelphia, Pennsylvania, USA

The pilot study by Xinning Su and Akira Midorikawa explores whether participants can retrieve genuinely neutral autobiographical memories using the cue words "home" and "study." Despite being asked to recall neutral memories, participants showed a positive memory bias, often recalling positive events instead. This study addressed an important question, but I have some concerns about the motivation behind the study and the methods. see below:

- The authors discuss how the repeated retrieval of positive autobiographical memories could potentially inhibit the recall of negative autobiographical memories. However, before delving into the specifics of their study and the underlying motivations (particularly the question of whether neutral and positive autobiographical memories contribute similarly to emotion regulation), it would be beneficial to examine prior research on the impact of retrieving neutral autobiographical memories. Specifically, it's essential to investigate whether the retrieval of neutral autobiographical memories can also inhibit the recall of negative memories.
- The authors should provide a clear rationale for the selection of specific cue words used in their study. This includes explaining why these particular words were chosen and how they align with the study's objectives. Additionally, it would be beneficial to consider incorporating a broader range of neutral cue words. This diversity could ensure a more comprehensive examination of the effects of neutral memory retrieval on the inhibition of negative memories and provide a more robust dataset for analysis.

- The use of self-rated valence to score autobiographical memories can introduce bias, as individuals may perceive and rate their memories subjectively. To mitigate this issue, it would be advantageous to have an independent rater evaluate the memories. This approach could provide a more objective assessment of the valence of memories, reducing the potential for self-report biases and enhancing the reliability of the study's findings.

- The statement "People generally retrieve autobiographical memories consistent with their self-image and beliefs, which tend to be positive" appears abruptly without prior mention of self-image or beliefs. It is crucial to introduce and discuss the concept of self-image and beliefs earlier in the manuscript. Providing a theoretical framework or citing relevant literature on how self-image and beliefs influence memory retrieval could offer context and support for this assertion. This would help integrate the statement more seamlessly into the discussion and strengthen the argument.

- The conclusion suggesting that future research should consider combining neutral and positive autobiographical memories by asking participants to recall neutral–positive memories is not well-supported by the current study. There is insufficient evidence to indicate that neutral and positive autobiographical memories function similarly, despite the observation that individuals tend to recall neutral memories more positively. To substantiate this conclusion, the authors should present stronger empirical evidence or theoretical justification demonstrating the potential benefits and similarities of combining these types of memories in emotion regulation and memory inhibition studies.

Is the work clearly and accurately presented and does it cite the current literature?

Yes

If applicable, is the statistical analysis and its interpretation appropriate?

Partly

Are all the source data underlying the results available to ensure full reproducibility?

Yes

Is the study design appropriate and is the work technically sound?

Partly

Are the conclusions drawn adequately supported by the results?

Partly

Are sufficient details of methods and analysis provided to allow replication by others?

Yes

Reviewer Expertise:

episodic memory, decision making

I confirm that I have read this submission and believe that I have an appropriate level of expertise to confirm that it is of an acceptable scientific standard, however I have significant reservations, as outlined above.

# Response

**Published:** 2024-10-15

**Authors:** Xinning Su

**Affiliations:** Psychology, Chuo University, Hachioji, Tokyo, Japan

**Response to Dr. Xinxu Shen:**

 We would like to express our sincere gratitude to the reviewers for your insightful comments on our manuscript. Your valuable comments have significantly contributed to enhancing the quality of our work.

****

**Comment 1:** The authors discuss how the repeated retrieval of positive autobiographical memories could potentially inhibit the recall of negative autobiographical memories. However, before delving into the specifics of their study and the underlying motivations (particularly the question of whether neutral and positive autobiographical memories contribute similarly to emotion regulation), it would be beneficial to examine prior research on the impact of retrieving neutral autobiographical memories. Specifically, it's essential to investigate whether the retrieval of neutral autobiographical memories can also inhibit the recall of negative memories.

**Response:** Thank you for this insightful comment. Previous research involving expressive writing has indeed explored the use of neutral content. For example, Sheerin et al. (2018) asked participants to write about neutral events, such as “what you did today”, and found that neutral writing also contributed to emotion regulation. However, due to paradigm differences, we did not include these studies in our current discussion. As for whether the retrieval of neutral autobiographical memories can inhibit the recall of negative memories, to our knowledge, no studies have specifically investigated this. However, a recent study found that retrieving neutral semantic memories can inhibit the recall of negative memories. We have added the following description to address this point (Paragraph 2 of the *Introduction*, lines 5-16):

*Regarding whether neutral memory retrieval can also inhibit the recall of negative memories, evidence from recent studies on semantic memory indicates that retrieving neutral semantic memories can lead to forgetting related negative memories (Greer et al., 2024). Considering that autobiographical and semantic memories share overlapping neural bases (Graham et al., 2003), and negative autobiographical memories are more strongly associated with emotional disorders (King et al., 2010; Krans et al., 2014), it is worth investigating whether the effects observed with neutral semantic memories might also apply to neutral autobiographical memories. Should neutral autobiographical memories function similarly to positive ones, this could extend the findings of Harris et al. (2010), offer new insights into the mechanisms of memory and emotion, and suggest alternative strategies for mitigating the effects of negative memories.*

**Comment 2:** The authors should provide a clear rationale for the selection of specific cue words used in their study. This includes explaining why these particular words were chosen and how they align with the study's objectives. Additionally, it would be beneficial to consider incorporating a broader range of neutral cue words. This diversity could ensure a more comprehensive examination of the effects of neutral memory retrieval on the inhibition of negative memories and provide a more robust dataset for analysis.

**Response:** Thank you for this pertinent feedback. We have added the following explanation to clarify why these particular words were chosen. (Paragraph 1 of the *Procedure*, lines 1-9)

*The cue words used by Harris et al. (2010) were “home” and “work”, likely chosen due to the wide age range of their participants (17–42 years old). For the cue word “work”, Harris et al. (2010) asked participants to recall autobiographical memories related to study or work. Given that all of our participants were college students of similar age, the cue words in our experiment were “home” and “study”, presented to each group. Participants were asked to generate five positive or five neutral autobiographical memories for each cue. Researchers informed the participants that the “study events” should be associated with study or work, whereas the “home events” should be related to home or family.*

 In addition, we have included your advice in the *Discussion* section of the revised version of the article. (Paragraph 3 of the *Discussion*, lines 1-5)

*This study had some limitations. First, since our ultimate aim is to extend the findings of Harris et al. (2010), the methods and cue words employed in this pilot study align with those described by Harris et al. (2010). While this approach is appropriate for the present study’s purposes, it would be beneficial to incorporate more neutral cue words to broaden the results further.*

**Comment 3:** The use of self-rated valence to score autobiographical memories can introduce bias, as individuals may perceive and rate their memories subjectively. To mitigate this issue, it would be advantageous to have an independent rater evaluate the memories. This approach could provide a more objective assessment of the valence of memories, reducing the potential for self-report biases and enhancing the reliability of the study's findings.

**Response:** Thank you for this insightful feedback. As you rightly pointed out, having an independent rater evaluate the memories is essential. Therefore, to address this limitation, we have added the following statement to the *Discussion *section. (Paragraph 3 of the *Discussion*, lines 5-11)

*This study had some limitations. ……Second, we adopted self-rating valence as per the methodology of Harris et al. (2010). However, self-ratings may be biased by various factors, including participants’ subjective interpretation of what constitutes a neutral event and their current mood ( Chiorri & Vannucci, 2024). Given the potential for subjective bias, future studies should incorporate both subjective self-ratings and objective assessments by independent raters to ensure a more accurate categorization of recalled events. This will allow for better verification of whether neutral memories are indeed perceived as neutral or if a bias toward positivity has occurred.*

**Comment 4:** The statement "People generally retrieve autobiographical memories consistent with their self-image and beliefs, which tend to be positive" appears abruptly without prior mention of self-image or beliefs. It is crucial to introduce and discuss the concept of self-image and beliefs earlier in the manuscript. Providing a theoretical framework or citing relevant literature on how self-image and beliefs influence memory retrieval could offer context and support for this assertion. This would help integrate the statement more seamlessly into the discussion and strengthen the argument.

**Response:** Thank you for your valuable feedback. In response to your suggestion, we have added the following note to the *Introduction*. (Paragraph 3 of the *Introduction*, lines 10-13)

*The self-memory system (Conway, 2005) suggests that autobiographical memory retrieval is influenced by an individual’s current beliefs, active goals, and self-image, which tend to be positive. As a result, people may unconsciously gravitate toward recalling positive autobiographical memories.*

**Comment 5:** The conclusion suggesting that future research should consider combining neutral and positive autobiographical memories by asking participants to recall neutral–positive memories is not well-supported by the current study. There is insufficient evidence to indicate that neutral and positive autobiographical memories function similarly, despite the observation that individuals tend to recall neutral memories more positively. To substantiate this conclusion, the authors should present stronger empirical evidence or theoretical justification demonstrating the potential benefits and similarities of combining these types of memories in emotion regulation and memory inhibition studies.

**Response: **Thank you for your insightful feedback. In response to your suggestion, we have added the following note to the *Discussion*. (The last paragraph of the *Discussion*)

*In conclusion, despite some limitations, we found that when limiting the number of recalls, participants tended to recall positive events even when asked to recall neutral autobiographical memories. Previous studies have found that retrieval of neutral semantic memories leads to forgetting of negative semantic memories (Greer et al., 2024), while retrieval of positive autobiographical memories leads to forgetting of negative autobiographical memories (Harris et al., 2010). Therefore, it is necessary to extend the findings of Harris et al. (2010) to examine whether neutral autobiographical memories can also inhibit the recall of negative memories. However, before extending the findings of Harris et al. (2010), we conducted this pilot study to determine whether genuinely neutral autobiographical memories can be retrieved without bias when prompted to recall neutral memories. Based on the results of this pilot study, it will be necessary to continue refining our understanding. Additionally, we can propose innovative ideas, such as exploring whether future work could integrate neutral and positive autobiographical memories, by asking participants to recall neutral–positive memories. We look forward to further research investigating whether, similar to the effects of retrieving positive memories (Burton & King, 2004; Burton & King, 2009; Harris et al., 2010), retrieving neutral–positive memories may also inhibit the recall of negative memories or enhance positive emotions. Such studies could provide valuable insights for developing emotional regulation strategies.*

 Once again, we sincerely thank you for your helpful comments and look forward to further comments on our manuscript.

# Reviewer report: Reviewer response for version 1

**DOI:** 10.5256/f1000research.160988.r294508

**Published:** 2024-07-16

**Authors:** Arkadiy Maksimovskiy

**Affiliations:** 1 Howard Hughes Medical Institute - Harvard Medical School, Boston, Massachusetts, USA

This pilot study tackled an important question by investigating whether participants were able to retrieve appropriate autobiographical memories when asked to recall a limited number of neutral or positive memories. ​ The study found that although participants were asked to recall neutral memories, they tended to recall positive memories. ​ This is intended to support the concept of positive memory bias. However, several limitations need to be addressed before the intended conclusion can be reached.

 1. The word ‘study’ was not used in the original procedure outlined by Harris (2010). Please explain why it was selected. Additionally, the terms ‘study’ and ‘home’ might overlap in our day and age. Please further explain the distinction.

 2. “In addition to autobiographical memories with positive or negative valence, memories can be neutral (D’Argembeau et al., 2003). It is unclear whether neutral and positive autobiographical memories contribute similarly to emotion regulation. However, before that question can be answered…”

 Please explain why neutral memories might contribute in the same way as positive memories. Why is this an important question? What are the mechanisms behind it?

 3. “However, most previous studies did not limit the number of recalls, which varied among events according to emotional valence. Notably, it is unclear whether participants can recall genuinely neutral memories when prompted if the number of recalls is limited.”

 It is unclear how neutral memory might be linked to the number of recalls.

 4. Figure 1: Why are two plots (box and whisker as well as bar graph) used to illustrate the same concept?

 Figure 1a: Please provide a distribution of scores for each participant. From the figure, it appears that ‘Positive study’ did not significantly differ from the neutral condition (contrary to the description), and the other positive category (i.e., ‘positive home’) barely (if at all) differed from neutral conditions. Please provide a distribution of individual scores and explain.

 5. If you’re asking participants to recall neutral events, but they recalled positive events, there is the possibility that participants did not see their ratings as neutral, which means the manipulation did not work (rather than a bias provided by authors). Please provide an explanation and further evidence as to how this is interpreted as a positive memory bias and whether any manipulation checks were done to ensure that neutral memories could be recalled.

 6. Thank you for providing original data. I ran a quick one-tailed t-test and am not seeing differences between the conditions (T-statistic: 1.82; P-value: 0.11). The code in python is below – please explain why this insignificant finding is consistent with your provided interpretation of the data:

 # Filter data for positive and neutral groups

 positive_data = data[data['group'] == 'positive']['valence_mean']

 neutral_data = data[data['group'] == 'neutral']['valence_mean']

 # Perform a t-test

 t_stat, p_value_two_tailed = stats.ttest_ind(positive_data, neutral_data)

 # Adjust the p-value for a one-sided test

 p_value_one_tailed = p_value_two_tailed / 2 if t_stat > 0 else 1 - p_value_two_tailed / 2

 # Display the results

 print(f'T-statistic: {t_stat}')

 print(f'P-value: {p_value}')

Is the work clearly and accurately presented and does it cite the current literature?

Yes

If applicable, is the statistical analysis and its interpretation appropriate?

No

Are all the source data underlying the results available to ensure full reproducibility?

Yes

Is the study design appropriate and is the work technically sound?

Partly

Are the conclusions drawn adequately supported by the results?

Partly

Are sufficient details of methods and analysis provided to allow replication by others?

Yes

Reviewer Expertise:

Cognitive neuroscience and memory research.

I confirm that I have read this submission and believe that I have an appropriate level of expertise to state that I do not consider it to be of an acceptable scientific standard, for reasons outlined above.

# Response

**Published:** 2024-10-15

**Authors:** Xinning Su

**Affiliations:** Psychology, Chuo University, Hachioji, Tokyo, Japan

**Respons to Dr. Arkadiy Maksimovskiy:**

 We would like to express our sincere gratitude to the reviewers for your insightful comments on our manuscript. Your valuable comments have significantly contributed to enhancing the quality of our work.

**Comment 1:** The word ‘study’ was not used in the original procedure outlined by Harris (2010). Please explain why it was selected. Additionally, the terms ‘study’ and ‘home’ might overlap in our day and age. Please further explain the distinction.

**Response:** Thank you for this pertinent feedback. The cue words used by Harris et al. (2010) were ‘home’ and ‘work’, which may have been due to the wide age range of their participants (17–42 years). For the cue word ‘work’, Harris et al. (2010) asked participants to recall autobiographical memories related to **study** or **work**. Given that our participants were similarly aged college students (19–24 years), the cue words in our experiment were ‘home’ and ‘study.’ The researcher told participants that the ‘study events’ should be related to study or work, and the ‘home events’ should be related to home or family. Therefore, we believe that although the words presented on the computer screen differed, in combination with the verbal instructions, we used the same cue words as Harris et al., (2010). For this reason, we have added the following description to the *Introduction* (The last paragraph of the *Introduction*, lines 3-6)

*Since our ultimate aim is to extend the findings of Harris et al. (2010), in the present pilot study, we adopted the same cue words as Harris et al. (2010), namely “home” and “study”, to investigate……*

In addition, we added the following description to the *Procedure*. (Paragraph 1 of the *Procedure*, lines 1-9)

*The cue words used by Harris et al. (2010) were “home” and “work”, likely chosen due to the wide age range of their participants (17–42 years old). For the cue word “work”, Harris et al. (2010) asked participants to recall autobiographical memories related to study or work. Given that all of our participants were college students of similar age, the cue words in our experiment were “home” and “study”, presented to each group. Participants were asked to generate five positive or five neutral autobiographical memories for each cue. Researchers informed the participants that the “study events” should be associated with study or work, whereas the “home events” should be related to home or family.*

**Comment 2:** “In addition to autobiographical memories with positive or negative valence, memories can be neutral (D’Argembeau et al., 2003). It is unclear whether neutral and positive autobiographical memories contribute similarly to emotion regulation. However, before that question can be answered…” Please explain why neutral memories might contribute in the same way as positive memories. Why is this an important question? What are the mechanisms behind it?

**Response:** Thank you for this insightful feedback. We appreciate the opportunity to clarify these important points.

 To address the first question: Why might neutral memories contribute in the same way as positive memories? We have added the following explanation in the revised manuscript. (Paragraph 2 of the *Introduction*, lines 5-8)

*Regarding whether neutral memory retrieval can also inhibit the forgetting of negative memories, evidence from recent studies on semantic memory indicates that retrieving neutral semantic memories can lead to forgetting related negative memories (Greer et al., 2024).*

 To address the second question: Why is this an important question? We have added the following explanation. (Paragraph 2 of the *Introduction*, lines 11-16)

*……it is worth investigating whether the effects observed with neutral semantic memories might also apply to neutral autobiographical memories. Should neutral autobiographical memories function similarly to positive ones, this could extend the findings of Harris et al. (2010), offer new insights into the mechanisms of memory and emotion, and suggest alternative strategies for mitigating the effects of negative memories.*

 To address the third issue: What are the mechanisms behind it? As well as link semantic and autobiographical memories. We have added the following clarification. (Paragraph 2 of the *Introduction*, lines 8-16)

*Considering that autobiographical and semantic memories share overlapping neural bases (Graham et al., 2003), and negative autobiographical memories are more strongly associated with emotional disorders (King et al., 2010; Krans et al., 2014), it is worth investigating whether the effects observed with neutral semantic memories might also apply to neutral autobiographical memories. Should neutral autobiographical memories function similarly to positive ones, this could extend the findings of Harris et al. (2010), offer new insights into the mechanisms of memory and emotion, and suggest alternative strategies for mitigating the effects of negative memories.*

**Comment 3:** “However, most previous studies did not limit the number of recalls, which varied among events according to emotional valence. Notably, it is unclear whether participants can recall genuinely neutral memories when prompted if the number of recalls is limited.” It is unclear how neutral memory might be linked to the number of recalls.

**Response:** Thank you for this pertinent feedback. Since our ultimate aim is to extend the findings of Harris et al. (2010), the pilot study conducted before this (i.e., the current study) needed to consider Harris’s procedures. Therefore, we limited the number of recalls and emotional valence. To clarify this issue, we added the following explanation.

*However, before we extend the findings of Harris et al. (2010), a pilot study is necessary to determine whether genuinely neutral autobiographical memories can be retrieved without bias when prompted to recall neutral memories.……* (Paragraph 3 of the *Introduction*, lines 1-3)

*However, most previous studies did not limit the number or valence of recalls. According to the procedure of Harris et al. (2010), participants were explicitly asked to recall a limited number of specified emotional events associated with each cue word. Since our ultimate aim is to extend the findings of Harris et al. (2010), in the present pilot study, we adopted the same cue words as Harris et al. (2010), namely “home” and “study”, to investigate whether appropriate autobiographical memories when asked to recall a limited number of neutral or positive memories.* (Paragraph 4 of the *Introduction*)

**Comment 4:** Figure 1: Why are two plots (box and whisker as well as bar graph) used to illustrate the same concept? Figure 1a: Please provide a distribution of scores for each participant. From the figure, it appears that ‘Positive study’ did not significantly differ from the neutral condition (contrary to the description), and the other positive category (i.e., ‘positive home’) barely (if at all) differed from neutral conditions. Please provide a distribution of individual scores and explain.

**Response:** Thank you for this insightful feedback. Based on your advice, we removed the bar graph, kept only the box-and-whisker plot and added the distribution of individual scores. (Figure 1)

**Comment 5:** If you’re asking participants to recall neutral events, but they recalled positive events, there is the possibility that participants did not see their ratings as neutral, which means the manipulation did not work (rather than a bias provided by authors). Please provide an explanation and further evidence as to how this is interpreted as a positive memory bias and whether any manipulation checks were done to ensure that neutral memories could be recalled.

**Response:** Thank you for raising this important point. As we followed Harris et al.’s (2010) methodology for the present study, we used self-rating valence and did not conduct explicit manipulation checks to confirm whether participants recalled neutral events as instructed. To make this clearer, we revised the *Procedure* as follows: (Paragraph 2 of the *Procedure*, lines 3-5)

*Then, as in Harris et al.’s (2010) methodology, after they had reported events, participants were asked to rate the valence of those events (1 = Very negative, 7 = Very positive).*

 However, as you point out, this is a limitation of the current study. we have therefore added the following clarification to the *Discussion *section. (Paragraph 3 of the *Discussion*, lines 5-11)

*This study had some limitations. ……Second, we adopted self-rating valence as per the methodology of Harris et al. (2010). However, self-ratings may be biased by various factors, including participants’ subjective interpretation of what constitutes a neutral event and their current mood (Chiorri & Vannucci, 2024). Given the potential for subjective bias, future studies should incorporate both subjective self-ratings and objective assessments by independent raters to ensure a more accurate categorization of recalled events. This will allow for better verification of whether neutral memories are indeed perceived as neutral or if a bias toward positivity has occurred.*

**Comment 6:** Thank you for providing original data. I ran a quick one-tailed t-test and am not seeing differences between the conditions (T-statistic: 1.82; P-value: 0.11). The code in python is below – please explain why this insignificant finding is consistent with your provided interpretation of the data:

 # Filter data for positive and neutral groups

 positive_data = data[data['group'] == 'positive']['valence_mean']

 neutral_data = data[data['group'] == 'neutral']['valence_mean']

 # Perform a t-test

 t_stat, p_value_two_tailed = stats.ttest_ind(positive_data, neutral_data)

 # Adjust the p-value for a one-sided test

 p_value_one_tailed = p_value_two_tailed / 2 if t_stat > 0 else 1 - p_value_two_tailed / 2

 # Display the results

 print(f'T-statistic: {t_stat}')

 print(f'P-value: {p_value}')

**Response:** Thank you for confirming our data and running the t-test. This t-test analysed whether there was a difference in self-reported valence ratings between the different valence groups (positive or neutral). The result was that there was no significant difference. This result is consistent with the ANOVA results we provided in the article. We conducted a 2 (valence group: positive/neutral) × 2 (cue: home/study) mixed ANOVA on self-reported valence ratings. The results revealed a nonsignificant main effect of the valence group. Therefore, thank you for validating our results. Although the result is negative (nonsignificant), it is still valuable to report for its potential contribution to understanding the topic.

 Once again, we sincerely thank you for your helpful comments and look forward to further comments on our manuscript.
